# Drug selection based on pan-genomics genetic features of *Mycobacterium tuberculosis*

**DOI:** 10.3389/fmicb.2025.1663069

**Published:** 2025-09-02

**Authors:** Xiangcheng Sun, Panpan Xu, Yun Shi, Ning Wang, Yan Li

**Affiliations:** ^1^Institute of Biopharmaceuticals, West China Hospital of Sichuan University, Chengdu, China; ^2^Laboratory of Liver Surgery, West China Hospital of Sichuan University, Chengdu, China

**Keywords:** *M* *. tuberculosis*, drug resistance, genetic diversity, DRMP, therapeutic and control strategies

## Abstract

Tuberculosis, caused by *Mycobacterium tuberculosis*, is a severe and persistent global public health issue, particularly exacerbated by the emergence of multidrug-resistant and extensively drug-resistant strains. This study employed pan-genomic approaches to analyze different strains with various resistance profiles, examining the diversity of bacterial genetic evolution in relation to mutations in resistance-related genes. The findings indicate that resistance-related genes are mostly core genes (94%), with a preference for base mutations closely associated with nonsynonymous mutations at resistance sites. Interestingly, while the majority of drugs induce positive selection in target genes, the tlyA gene under the influence of amikacin (AMI) undergoes passive selection. Cluster analysis of target genes suggests consistency between SNP clusters and drug-resistant clusters, revealing a strong correlation between bacterial evolutionary branches and resistance profiles. Consequently, based on pan-genome evolutionary characteristics, we identified the drug-resistant mutation pattern (DRMP) that can serve as a molecular fingerprint and indicator for drug sensitivity, aiding in the assessment and guidance of drug selection for treating different strains and the formulation of individualized treatment plans. This research not only enhances our understanding of the mechanisms of drug resistance in *M. tuberculosis* but also offers new perspectives for the development of new drugs, which is crucial for global tuberculosis control.

## 1 Introduction

Tuberculosis (TB), a historically pervasive and enduring infectious disease, continues to pose a formidable challenge to global public health (Dheda et al., 2014; Shaku and Bishai, 2022; Farhat et al., 2024; Shu and Liu, 2024), remaining one of the leading causes of mortality worldwide. Despite the continuous optimization of TB prevention and control strategies over the past few decades, the emergence of drug-resistant *M. tuberculosis* has significantly undermined these efforts (Ehrt et al., 2018; Koch and Mizrahi, 2018; Farhat et al., 2024; Shu and Liu, 2024). The appearance of multidrug-resistant (MDR) and extensively drug-resistant (XDR) strains (Wulandari et al., 2024) has particularly limited treatment options, prolonged treatment durations, increased costs, and substantially diminished treatment efficacy (Gandhi et al., 2010; Singh et al., 2020; Shitikov and Bespiatykh, 2023).

The rapid advancement of molecular biology technologies, especially the widespread application of high-throughput sequencing techniques, has markedly enhanced our understanding of the drug resistance mechanisms in *M. tuberculosis* (Hanif and Arora, 2022). Researchers have identified various gene mutations associated with drug resistance in *M. tuberculosis*. These mutations involve genes critical for the clinical treatment of TB, such as: mutations in the embB and embC genes associated with ethambutol (EMB) resistance (Sreevatsan et al., 1997; Ramaswamy et al., 2000; Lee et al., 2004; Srivastava et al., 2006; Srivastava et al., 2009); specific mutations in the inhA and katG genes related to isoniazid (INH) and ethionamide (ETH) resistance (Lee et al., 2000; Morlock et al., 2003; Lavender et al., 2005; Sekiguchi et al., 2007); mutations within the ethA and inhA structural genes also linked to ETH resistance (Lavender et al., 2005); and high double-point mutations in the gyrA gene indicating the emergence of fluoroquinolone resistance (Aubry et al., 2006; Shi et al., 2006), among others. The researchers have found that the genetic diversity of *M. tuberculosis* is crucial for its evolutionary selection under drug pressure, understanding these genetic evolutionary patterns is significantly meaningful for preventing drug resistance and guiding medication selection (Müller et al., 2013; Eldholm et al., 2014; Cohen et al., 2015; Gagneux, 2018). However, despite some progress in previous research, a systematic understanding of how *M. tuberculosis* evolves under different pressures is still lacking.

Therefore, this study employed pan-genomic analysis techniques on strains from different sources and with varying drug resistance profiles to comprehensively explore the genetic diversity of *M. tuberculosis* and its association with drug resistance. By examining the evolutionary trajectory of drug-resistant related genes, we aim to uncover how these genetic variations impact the drug resistance of strains. This research not only aids in deepening our comprehension of the drug resistance mechanisms in *M. tuberculosis*, but also showcases patterns of drug-related mutations, offering a scientific basis for prevention and control strategies and facilitating the implementation of precision treatment.

## 2 Materials and methods

### 2.1 Data collection and processing

We obtained a diverse collection of genomic data from two sources within the NCBI database, which cover the last two decades from August 2004 to May 2024. Firstly, we acquired raw sequencing reads from 669 sequenced *M. tuberculosis* isolates available through the NCBI Sequence Read Archive (SRA).^1^ These data provided a broad representation of the genetic diversity present in *M. tuberculosis* strains worldwide. Additionally, we included an extra 470 fully assembled *M. tuberculosis* isolates from the NCBI Assembly database^2^ to enrich our analysis with fully annotated genomic sequences (Supplementary Table S1). Sequencing reads were quality-trimmed with Trimmomatic v0.39 (Bolger et al., 2014) to remove adapters and low-quality bases. High-quality reads were aligned to the *M. tuberculosis* H37Rv reference genome (NCBI: NC_000962.3) using BWA-MEM v0.7.18 (Jung and Han, 2022) with default parameters. Resulting alignments (SAM format) were converted to sorted BAM files using SAMtools v1.19.2 (Danecek et al., 2021) and subsequently transformed into FASTQ format using BEDTools v2.31.1 (bamtofastq) (Quinlan and Hall, 2010) with default settings. *De novo* genome assembly was performed on processed reads using SOAPdenovo2 v2.41 (Luo et al., 2012) with optimized parameters -K 127 -p 16 -F -R -u (asm_flags = 3, rank = 1; other parameters default). Scaffolding leveraged paired-end read information, and internal gaps were closed using GapCloser v1.12 (Luo et al., 2012) with parameters -l 150 -p 30 -t 16. Assembly completeness was assessed with BUSCO v5.4.5 (Manni et al., 2021) using the bacteria_odb10 lineage dataset^3^ in genome mode with parameters -m geno -c 16 --long.

### 2.2 Gene annotation

Reference protein-coding sequences (CDSs) from *M. tuberculosis* H37Rv (NCBI: NC_000962.3) were extracted from GenBank annotations, converted to nucleotide FASTA format with retention of original locus tags and functional descriptions, and compiled into a custom BLAST database using makeblastdb v2.14.0 (Camacho et al., 2009). This database was filtered to exclude pseudogenes and CDSs <100 bp. Orthologous genes were identified via BLASTn (Camacho et al., 2009) alignment against the target genome assembly under stringent parameters: *E*-value ≤1 × 10^−5^, minimum nucleotide identity 70%, and query/subject coverage ≥80% (-*E*-value 1 × 10^−5^ -perc_identity 70 -qcov_hsp_perc 80). Matches fulfilling all criteria inherited H37Rv-derived locus tags and functional annotations.

### 2.3 Pan-genome analysis

Pan-genome analysis was performed using IPGA (integrated prokaryotes genome and pan-genome analysis) (Liu et al., 2022), a robust tool for prokaryotic genome and pan-genome analysis. Input genome files underwent automatic quality control, retaining genomes with >90% completeness and <5% contamination for downstream analysis. Genes were predicted in quality-controlled genomes using IPGA, and the resulting predictions served as input for the pan-genome analysis module. Within this module, the integrated software packages PanOCT (Inman et al., 2019), OrthoMCL (Li et al., 2003), Roary (Page et al., 2015), panX (Ding et al., 2017), OrthoFinder (Emms and Kelly, 2015), Panaroo (Tonkin-Hill et al., 2020), and PPanGGoLiN (Gautreau et al., 2020) were employed with the following parameters: Identity = 70, Ratio (core) = 0.95, Support = −1. Pan-genome profiles generated by the different tools were subsequently processed by the optimal selection module to identify the highest-quality pan-genome profile. To systematically characterize the potential for horizontal gene transfer within our assembled genomes, we identified and annotated mobile genetic elements (MGEs) using the mobileOG-db module (Brown et al., 2022) integrated within the Proksee v1.1.3 platform (Grant et al., 2023). This approach leveraged the curated mobileOG database, a comprehensive resource specifically designed for MGE annotation and encompassing protein families associated with plasmids, bacteriophages, and integrative elements (including functions such as conjugation, transposition, replication, and integration/excision). Assembled genomic sequences (FASTA format) were analyzed within Proksee, where the module employed HMMER3 (hmmscan) (Finn et al., 2011) to query predicted protein sequences against the database’s profile hidden Markov models (HMMs). Significant hits were filtered using default thresholds (*E*-value ≤ 1 × 10^−5^, alignment coverage) to assign functional annotations and categorize MGE-associated genes. Putative MGEs were inferred based on the co-localization and clustering of multiple annotated genes encoding related functions. Finally, customizable circular genome plots were generated using Proksee integrated visualization capabilities to depict the genomic context, location, and distribution of identified MGE-associated genes relative to other features, exporting these as high-resolution vector graphics (SVG) for publication.

### 2.4 SNP identification and analysis

Single nucleotide polymorphism (SNP) identification was performed using an integrated pipeline with BCFtools v1.15.1 (Danecek et al., 2021) and GATK v4.2.6.1 (Van der Auwera et al., 2013) for variant calling, followed by error correction and lineage-specific variant annotation using TB-gen v0.6.1.^4^ Genetic clusters were defined by grouping isolates exhibiting a pairwise SNP distance ≤12, calculated from whole-genome SNP matrices generated with Parsnp v2.0.5 (Kille et al., 2024). For each cluster, the mutation rate was calculated as the average number of SNPs per site per isolate, derived from high-quality SNP calls (QUAL > 30, DP > 10, GQ > 20) within all isolates of the cluster. This rate was computed by dividing the total number of identified SNPs by the product of the number of isolates in the cluster and the core genome length (4.1 Mb). To identify cluster-specific SNP loci, a merged multi-sample VCF file (generated using BCFtools merge) containing variants from all clusters served as input. Cluster-specific loci were defined as genomic positions harboring variants present exclusively in one cluster and absent in all others. Variants private to each cluster were isolated using BCFtools isec and subsequently validated against the TB-gen database to exclude known lineage-defining markers, ensuring the identified uniqueness was specific to the cluster context.

### 2.5 Predicted drug resistance

First, we used the TB-AMRpred pipeline^5^ (Pal and Mohanty, 2025) to predict antimicrobial drug resistance in *M. tuberculosis* based on whole genome sequences. Then, we combined this with the tbAnnotator pipeline^6^ for analysis. By running the tbAnnotator.py script, we queried a database constructed from literature-based drug susceptibility experimental data and scored new SNPs, generating text in json format. To more intuitively display the predicted drug resistance results, we further regenerated HTML reports using the htmlReportRegenerator.py script.

### 2.6 Whole-genome phylogenetic reconstruction

Whole-genome phylogenies were inferred using RealPhy v1.13 (Bertels et al., 2014) (parameters: -minlen 50, -minqual 20) from high-quality genome assemblies (FASTA format; assessed with CheckM v1.2.3 (Parks et al., 2015): completeness >95%, contamination <5%). This reference-guided approach generated a multiple sequence alignment incorporating SNPs identified *de novo* and via reference mapping. Gap-rich sites (>90% gaps) were removed using trimAl v1.5.0 (-gt 0.1) (Capella-Gutiérrez et al., 2009). Maximum-likelihood phylogenetic reconstruction was performed with IQ-TREE v2.3.4 (Minh et al., 2020), employing the ModelFinder-Plus algorithm (-m MFP + ASC) (Kalyaanamoorthy et al., 2017) to select the optimal substitution model while accounting for ascertainment bias (ASC). Branch support was assessed using 1,000 ultrafast bootstrap replicates (UFBoot; -B 1000 --bnni) and the Shimodaira-Hasegawa approximate likelihood ratio test (SH-aLRT; -alrt 1,000); clades with UFBoot ≥95% and SH-aLRT ≥80% were considered well-supported. The entire workflow was replicated three times to confirm topological consistency. Final tree visualization and annotation utilized iTOL v6 (accessible at https://itol.embl.de/) (Letunic and Bork, 2021).

### 2.7 Amino acid mutation frequency quantification

Amino acid mutation frequencies were determined by calculating the percentage of alterations observed at mutated positions in analyzed resistance gene sites. A “gain” event denotes the introduction of a specific amino acid at a position where it was previously absent, while a “loss” event indicates the replacement of a specific amino acid originally present at that position. The gain frequency (Freq_gain_) and loss frequency (Freq_loss_) for each amino acid were calculated as:


$${\text{Freq}}_{\text{gain}/\text{loss}}=\frac{\sum {\text{Count}}_{\text{gain}/\text{loss}}}{\sum {\text{Total}}_{\text{mutated}}}$$

where: $\sum {\text{Count}}_{\text{gain}/\text{loss}}$ = Total number of gain/loss events across all sequences, $\sum {\text{Total}}_{\text{mutated}}$ = Total number of mutated amino acid positions in all sequences. This metric reflects the proportion of gain or loss events per amino acid relative to all observed mutations.

### 2.8 Evolutionary selection pressure on drug resistance-associated genes

To assess the selection pressure acting on these protein-coding genes, we extracted the precise genomic coordinates of the target resistance genes (the 31 genes identified through resistance analysis) from species-specific annotation files using an awk script, generating corresponding BED-format files. Nucleotide coding sequences (CDS) were then batch-extracted from the assembled genomes based on these coordinates using bedtools getfasta -s -name+. These CDS sequences were subsequently translated into amino acid sequences in batch using a custom Python script employing the Biopython library. Finally, the ratios of non-synonymous (*K*_a_) to synonymous (*K*_s_) substitution rates (*K*_a_/*K*_s_) were calculated batch-wise using the ParaAT pipeline (Zhang et al., 2012), where sequence alignment was performed by MAFFT v7.526 using default parameters, and *K*_a_ and *K*_s_ values were computed by *K*_a_*K*_s__Calculator v3.0 (Zhang, 2022) using default parameters. *K*_a_/*K*_s_ ratios were interpreted as follows: *K*_a_/*K*_s_ > 1 indicates positive selection (favoring fixation of amino acid-altering mutations); *K*_a_/*K*_s_ = 1 indicates neutral evolution (random fixation of mutations); *K*_a_/*K*_s_ < 1 indicates purifying selection (removal of amino acid-altering mutations). We visualized the results by plotting *K*_a_/*K*_s_ density plots using ggplot2 v3.5.1 in R v4.2.1.

### 2.9 Cluster analysis

Based on the resistance stratification data, we utilized unsupervised clustering analysis to categorize a collection of 600 strains. To determine the stable number of clusters, we employed the ConsensusClusterPlus22 R package, performing clustering analyses across all groups through 1,000 iterations using the KM hierarchical clustering algorithm. Additionally, we utilized PCA (principal component analysis) to further validate the stability of the classifications. Subsequently, we used the VCF2PCACluster (He et al., 2024) to perform PCA and clustering analysis on the SNP (single nucleotide polymorphism) data.

### 2.10 Muti-gene phylogeny

Coding sequences (CDSs) of 31 resistance genes were individually aligned using MAFFT v7.505 with the --auto parameter in PhyloSuite v1.2.3 (Zhang et al., 2020). Poorly aligned regions were trimmed using trimAl v1.4 with the -automated1 heuristic to preserve reliable phylogenetic signal. The trimmed alignments were then concatenated into a super matrix using PhyloSuite’s integrated concatenation tool. Optimal partitioning schemes (by gene and codon position) and nucleotide substitution models were determined under the Bayesian information criterion (BIC) using PartitionFinder v2.1.1 (Lanfear et al., 2017), employing a greedy search algorithm to evaluate model combinations. Maximum likelihood (ML) phylogenies were reconstructed with IQ-TREE v2.3.4, applying partition-specific substitution models. Topological robustness was assessed via 10,000 ultrafast bootstrap (UFBoot) replicates and by evaluating 100 distinct random starting trees to ensure consistency. Final trees were visualized and annotated in iTOL v6.

### 2.11 Differential analysis of SNPs

We constructed a matrix of SNPs and refined it meticulously to ensure data accuracy and consistency. During the matrix establishment, we set a criterion: if a mutation occurred at a particular locus within the sample, that locus was labeled as 1; if no mutation occurred, it was labeled as 0. Subsequently, we conducted a comprehensive comparative analysis across different cluster types to identify which SNPs had a mutation rate exceeding 90% in each cluster type and further filtered out SNP loci unique to each category. These filtered loci are referred to as drug-resistant mutation pattern (DRMP).

### 2.12 Statistical analysis

For statistical analysis and graphical generation, we utilized R Project v4.0.2 (accessible at https://www.r-project.org/). In terms of text processing, we employed Perl v5.15 (available at https://www.perl.org/) and Python v3.10 (accessible at https://www.python.org/). To calculate the correlation between gene mutation bases and amino acid usage and evolutionary rates, we applied the Spearman’s rank correlation analysis method. For drawing and beautifying the evolutionary tree, we used iTOL v6 (accessible at https://itol.embl.de/) (Letunic and Bork, 2021).

## 3 Results

### 3.1 Landscape on the *Mycobacterium tuberculosis* pan-genome

**Figure 1:**
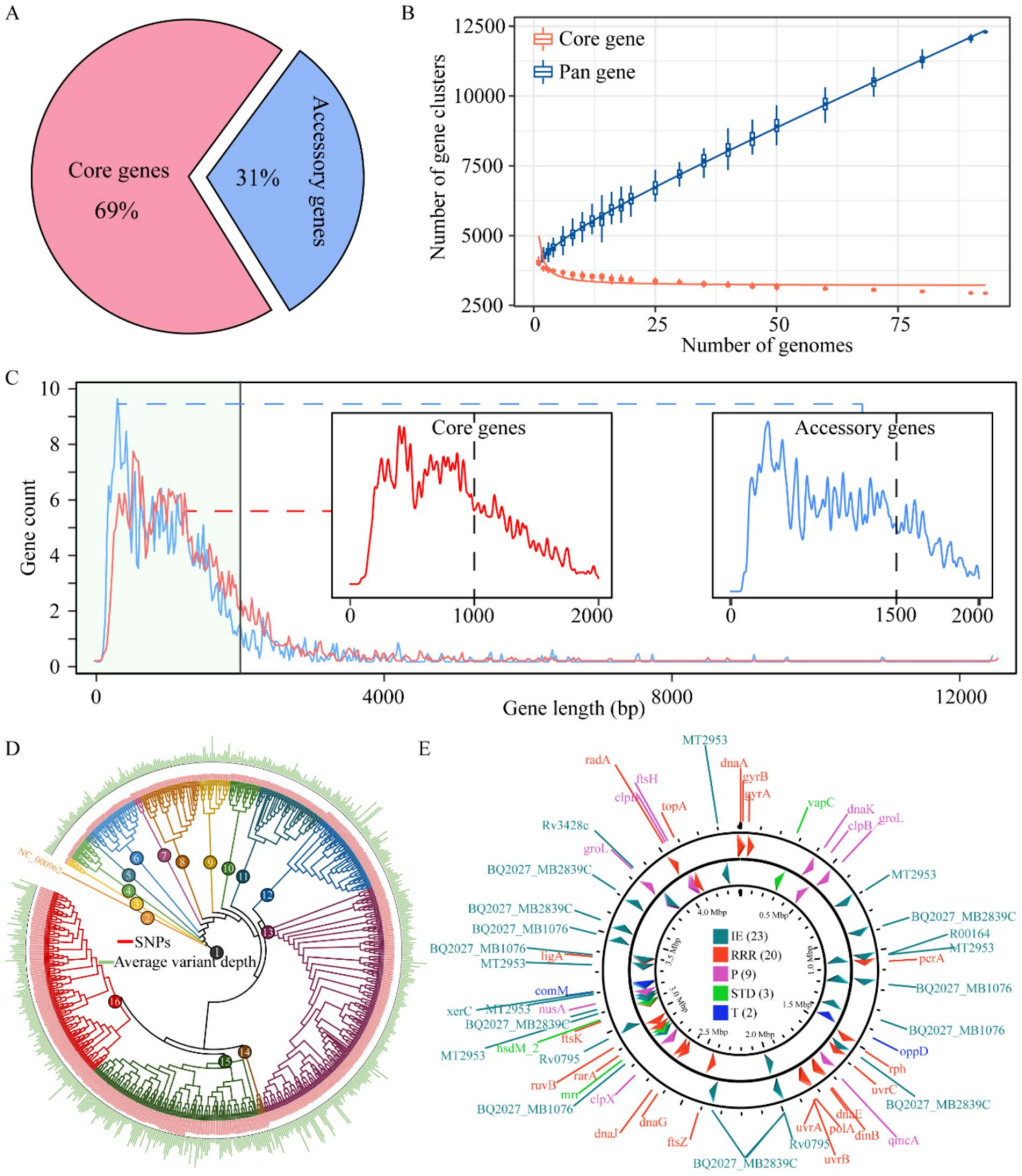
Pan-genome mapping of *M. tuberculosis*. Proportion of core and accessory genes among *M. tuberculosis* **(A)**, plot of changes in the number of pan- and core gene clusters with the addition of *M. tuberculosis* **(B)**. Distribution of gene sequence lengths in the *M. tuberculosis* pan-genome, x-axis: gene sequence length (bp), y-axis: number of genes **(C)**. Phylogenetic relationships among *M. tuberculosis* strains based on whole-genome variation **(D)**. Spatial distribution of mobile genetic elements in the *M. tuberculosis* pan-genome **(E)**. IE, integration/excision, RRR, replication/recombination/repair; P, phage; STD, stability/transfer/defense; T, transfer.

We constructed a comprehensive pan-genome of *M. tuberculosis* by assembling 1,000 complete genomes of this pathogen. Using the IPGA scoring system, we identified that panX exhibited the most optimal performance in the pan-genome analysis of *M. tuberculosis*. The results revealed a striking imbalance in gene distribution: core genes constituted the overwhelming majority (69%) of the total gene complement, while accessory genes accounted for only 31%. This underscores the substantial core genome shared among the analyzed strains (Figure 1A). Additionally, the number of pan-gene clusters increased to 12,295, whereas the number of core gene clusters decreased to 2,935. Meanwhile, the curve began to plateau with additional strains, indicating that further strain addition had a minimal impact on defining the core genome (Figure 1B). Upon further statistical analysis of the gene sequence length distribution within the pan-genome, we observed a decrease in the number of genes as the gene length increased. Most genes were found to be under 2,000 bp in length. Notably, core genes primarily consisted of shorter sequences (under 1,000 bp), resulting in a smoother curve. In contrast, accessory genes were more prevalent among longer sequences (1,500 bp), leading to a more fluctuating curve (Figure 1C). Based on the phylogenetic inference using whole-genome SNPs, we were able to classify these genomes into 16 distinct clusters (Figure 1D). Furthermore, our analysis of mobile genetic elements involved in constructing the pan-genome revealed that integration/excision (IE) was the most frequently annotated, with 23 occurrences. This was followed by replication/recombination/repair (RRR) (20), phage (P) (9), and stability/transfer/defense (STD) (3), transfer (T) being the least frequent of 2 (Figure 1E). Overall, we successfully constructed the pan-genome of *M. tuberculosis*, providing valuable insights into its genetic diversity and evolutionary history. This achievement highlights the power of pan-genome analysis in elucidating the complex genomic landscape of infectious diseases.

### 3.2 Pan-genomic variation in *Mycobacterium tuberculosis*

**Figure 2:**
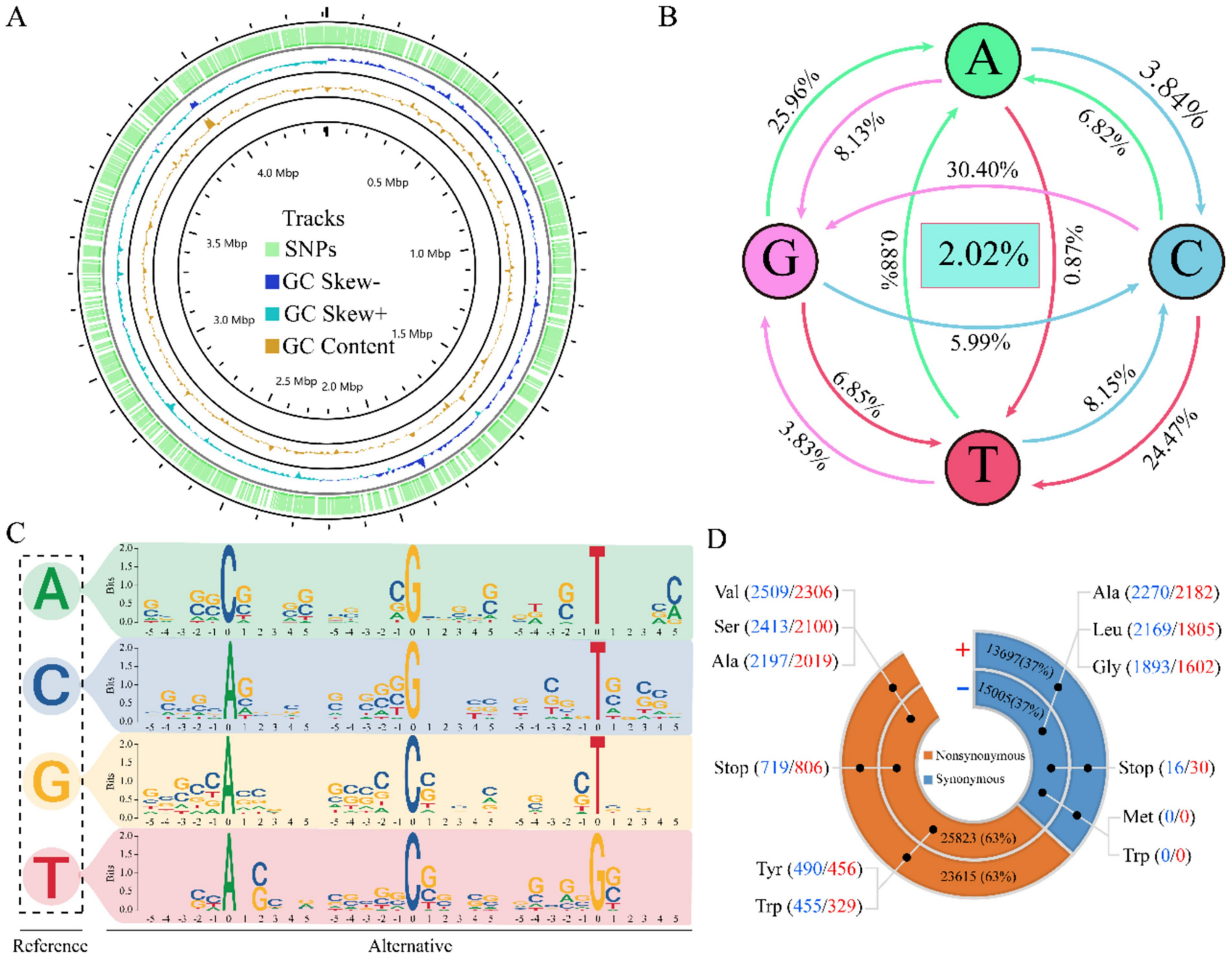
Panoramic analysis of variation. Distribution of SNPs and GC content at the pan-genomic level **(A)**. Statistical map of mutant base transitions **(B)**. Motif plots of sequence features for each of the five bases before and after the mutant site are depicted **(C)**. Chart of amino acid change results **(D)**.

To thoroughly investigate the *variation* of *M. tuberculosis*, we conducted an extensive analysis of the distribution of SNPs across the entire genome. Our findings indicate a lack of pronounced mutation hotspots; however, they suggest that genetic diversity arises ubiquitously throughout the genome rather than being concentrated in specific genomic segments (Figure 2A), upon statistical analysis of all base mutations, it became evident that *M. tuberculosis* exhibits a striking level of conservatism at the nucleotide level, with 98.08% stability observed. Only a minuscule fraction, 2.02% of bases, were found to be mutated. This high degree of genetic stability suggests that most regions of the *M. tuberculosis* genome are under strong selective pressure to maintain function. Among the identified mutations, the transition from cytosine (C) to guanine (G) was the most prevalent, accounting for 25.96% of all mutations. This was followed closely by the transition of guanine (G) to adenine (A), which constituted 24.47% of mutations. In contrast, transversions between adenine (A) and thymine (T) were exceedingly rare, with A to T mutations occurring at a frequency of 0.87% and T to A mutations at 0.88%. This pronounced asymmetry in substitution types highlights a fundamental constraint or bias in the mutagenic processes shaping *M. tuberculosis* evolution (Figure 2B). To investigate sequence preferences influencing mutagenesis, we generated position-weighted sequence logos centered on each nucleotide (A, C, G, T) with 5-bp flanking contexts. Motif analysis consistently revealed significant enrichment of C/G bases immediately adjacent to mutated sites across all central nucleotides (*p* < 0.001, Fisher’s exact test). This conserved pattern suggests that C/G dinucleotides may facilitate mutagenesis by stabilizing local structural dynamics or recruiting specific protein interactors (Figure 2C). To elucidate the potential impact of these mutations on protein sequence and function, we statistically analyzed the translational changes subsequent to the mutations. We counted the number of amino acid changes encoded by the mutated sites and discovered that synonymous (63%) and nonsynonymous mutations (37%) occurred in similar proportions in both the positive and negative strands. Within synonymous mutations, the amino acid alanine (Ala) was most frequently unaffected. In the realm of nonsynonymous mutations, valine (Val) was the amino acid most commonly subjected to change. Notably, tryptophan (Trp) exhibited only nonsynonymous mutations, suggesting its critical role in protein structure or function. Additionally, we observed an increase in the number of termination codons resulting from the mutations, which could have significant implications for gene expression and pathogenicity (Figure 2D). These insights not only enhance our understanding of the genetic diversity and evolution of *M. tuberculosis* but also have important implications for developing targeted therapeutic strategies against this globally significant human pathogen.

### 3.3 Drug resistance-related genes of *Mycobacterium tuberculosis*

**Figure 3:**
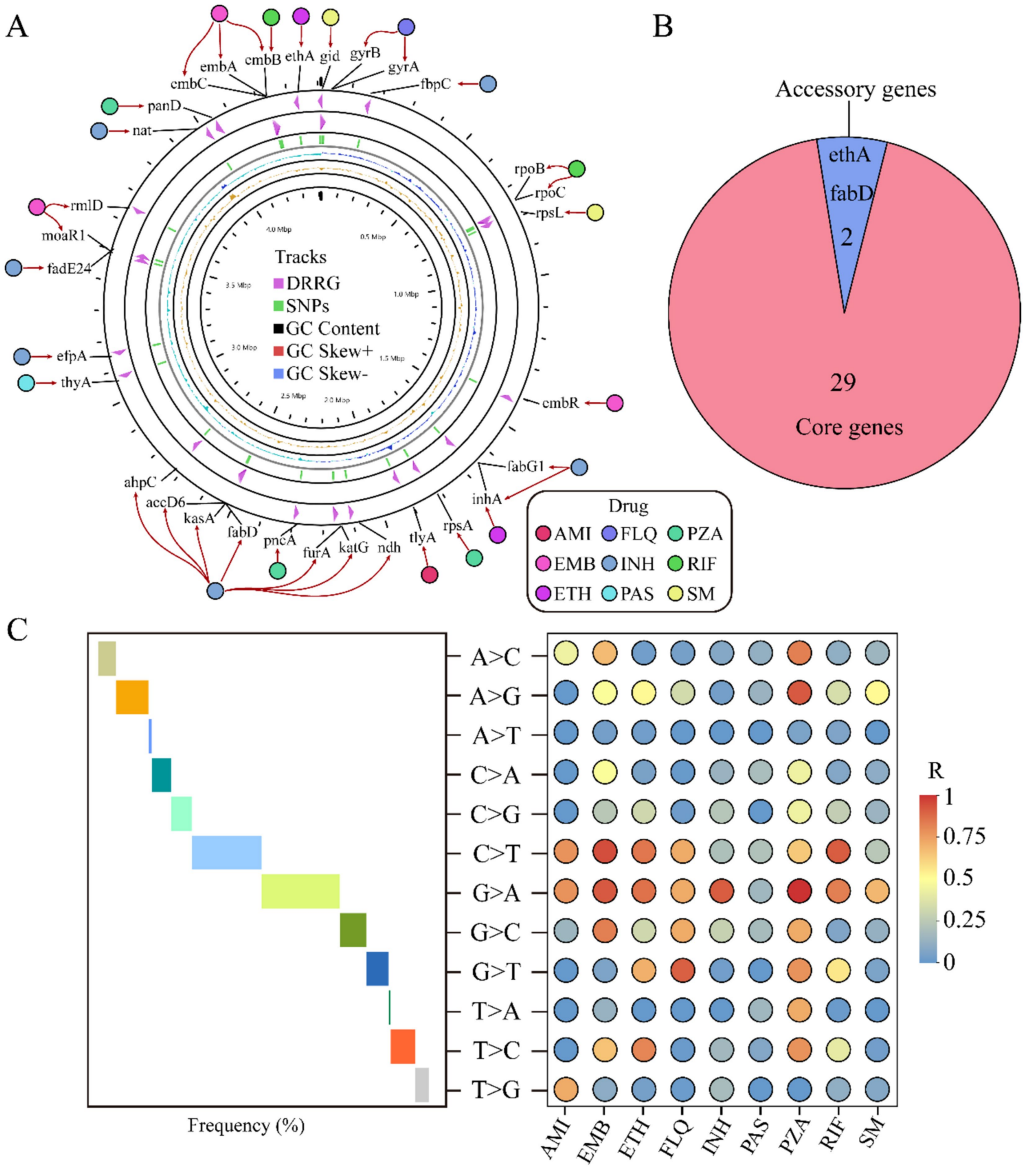
Analysis of drug resistance-related genes. The circular diagram displays the distribution of drug resistance-related genes and their relationship to the nine drugs. From the outside to the inside, the diagram indicates: drug names, drug resistance-related genes, distribution of SNPs, GC content, positive strand GC content, and negative strand GC content **(A)**. Pie chart shows the distribution of drug resistance-associated genes across the pan-genome **(B)**. Trend chart of mutation base type (middle) and correlation between nine different drug resistances (right) **(C)**. AMI, amikacin; EMB, ethambutol; ETH, ethionamide; FLQ, fluoroquinolones; INH, isoniazid; PAS, para-aminosalicylic acid; PZA, pyrazinamide; RIF, rifampicin; SM, streptomycin.

Our comprehensive investigation into genes associated with drug resistance uncovers an intriguing distribution pattern: these genes are dispersed across both the positive and negative strands of the genome. Among them, genes like embB stand out for their capacity to resist multiple drugs. Moreover, our analysis reveals a complex interplay where multiple genes can collaboratively contribute to the resistance against a single drug (Figure 3A). A pan-genomic examination of these pivotal genes discloses that the majority are classified as core genes, underscoring their fundamental role in the organism’s survival. Only two genes, ethA and fabD, were characterized as auxiliary, suggesting a more specialized function (Figure 3B). The mutation rate among these genes was remarkably low at 1%, with the predominant mutation being a guanine (G) transitioning to adenine (A). This specific G to A mutation was the most frequent, highlighting a potential hotspot for genetic alterations impacting drug resistance. Furthermore, we analyzed the correlation between the types of variant bases and resistance to nine different antimicrobial drugs. Our results revealed a positive correlation between the occurrence of base mutations and the resistance levels observed for these drugs. Interestingly, when evaluating the resistance conferred by different mutated bases, we found that mutations exhibiting the least resistance correlation were those associated with para-aminosalicylic acid (PAS). This implies that PAS remains relatively efficacious even against strains harboring certain mutations, potentially due to the drug’s unique mechanism of action or the types of mutations that arise in its presence. In contrast, mutations showing a higher resistance correlation were those associated with pyrazinamide (PZA). These insights into the nuanced relationships between mutated bases and drug resistance have important implications for understanding the evolution of drug-resistant strains. They also emphasize the need for continuous surveillance of mutational patterns to predict and counteract the emergence of resistant phenotypes. Moreover, this information can guide the development of more robust therapeutic strategies that are less susceptible to existing resistance mechanisms, ultimately improving clinical outcomes in the battle against multidrug-resistant infections (Figure 3C). These findings underscore the diverse mechanisms by which different drugs are rendered ineffective due to genetic changes. This insight not only advances our understanding of drug resistance at the genomic level but also paves the way for more targeted and effective strategies to combat drug-resistant strains.

### 3.4 The relationship between gene mutations and the rate of gene evolution

**Figure 4:**
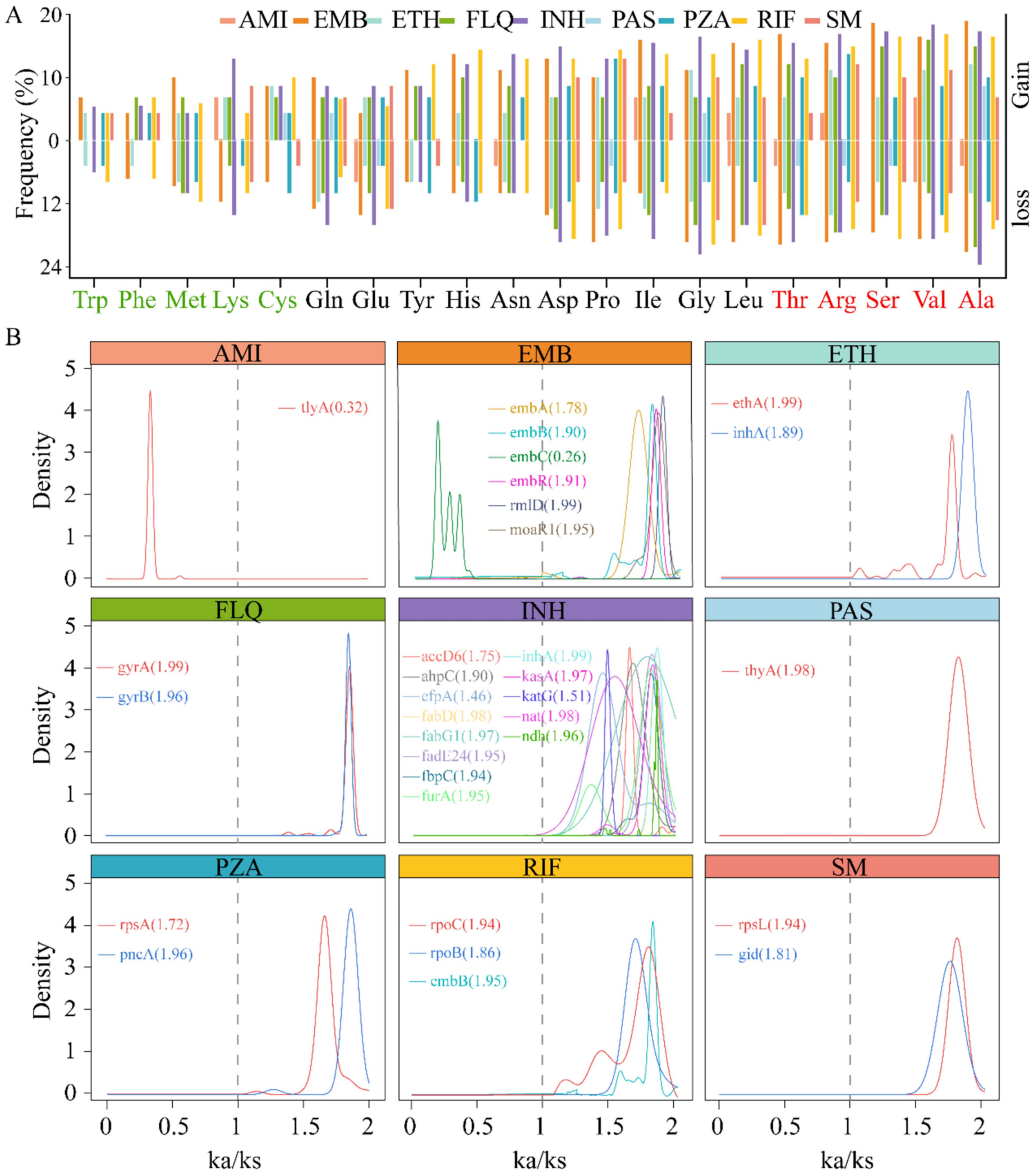
Correlation between gene mutation and gene evolutionary rate. The histogram displays the changes in amino acid (AA) usage frequency of nine drug resistance-related genes caused by gene mutations. The five most frequently used AAs are marked in red on the right, while the five least frequently used AAs are indicated in light green on the left **(A)**. Density plot of the rate of evolution of resistance genes (*K*_a_/*K*_s_ ratio), the lines represent the corresponding genes, with the values indicating the median. *K*_a_/*K*_s_ > 1 indicates that the gene is under positive selection, *K*_a_/*K*_s_ = 1 suggests neutral evolution of the gene, and *K*_a_/*K*_s_ < 1 implies that the gene is undergoing purifying selection **(B)**.

The dynamics of gene mutations significantly influence the evolutionary trajectory of protein sequences. These evolutionary changes typically encompass the acquisition and deletion of amino acids (AAs), which can profoundly affect protein function and structural stability. In our study, we conducted an in-depth analysis of the AA variations encoded by genes associated with drug resistance. Our study findings reveal that, among the resistance genes examined, the proportion of the AA variant is significantly higher than other types, notably, alanine (Ala), valine (Val), serine (Ser), arginine (Arg), and threonine (Thr) were high variability, suggesting a possible correlation between the frequency of these residues and the adaptive advantage conferred by resistance genes. Conversely, our analysis also identified a set of AAs that appear to be more stable in these genes. Including Trp, phenylalanine (Phe), methionine (Met), lysine (Lys), and cysteine (Cys) (Figure 4A). This divergence in AA usage may reflect functional constraints or selective pressures unique to the resistant phenotypes. To explore the impact of mutations on the evolutionary rate of genes, we examined the ratio of nonsynonymous (*K*_a_) to synonymous (*K*_s_) substitutions, a metric commonly used to infer selection pressures acting on protein-coding genes. Strikingly, we observed varied *K*_a_/*K*_s_ ratios across different resistance genes, indicating heterogeneity in their evolutionary trajectories. Remarkably, the majority of the resistance genes exhibited signatures of positive selection, indicated by *K*_a_/*K*_s_ ratios greater than 1. This pattern suggests that these genes are evolving under pressures that favor new variants, potentially due to environmental challenges such as exposure to antimicrobial agents. In stark contrast, only two genes, tlyA and embC, showed signs of purifying selection with *K*_a_/*K*_s_ ratios of 0.3 and 0.26, respectively (Figure 4B). Purifying selection, characterized by *K*_a_/*K*_s_ ratios less than 1, operates to remove deleterious variants from the population, implying that most mutations in these genes are likely to be harmful and thus eliminated over time. Taken together, these results provide compelling evidence that the evolution of drug resistance in bacterial populations is a complex process influenced by both the accumulation of advantageous mutations and the elimination of detrimental ones. This deeper understanding of genetic variation and its impact on evolutionary dynamics can inform strategies to mitigate the spread of antimicrobial resistance.

### 3.5 Cluster analysis of *Mycobacterium tuberculosis*

**Figure 5:**
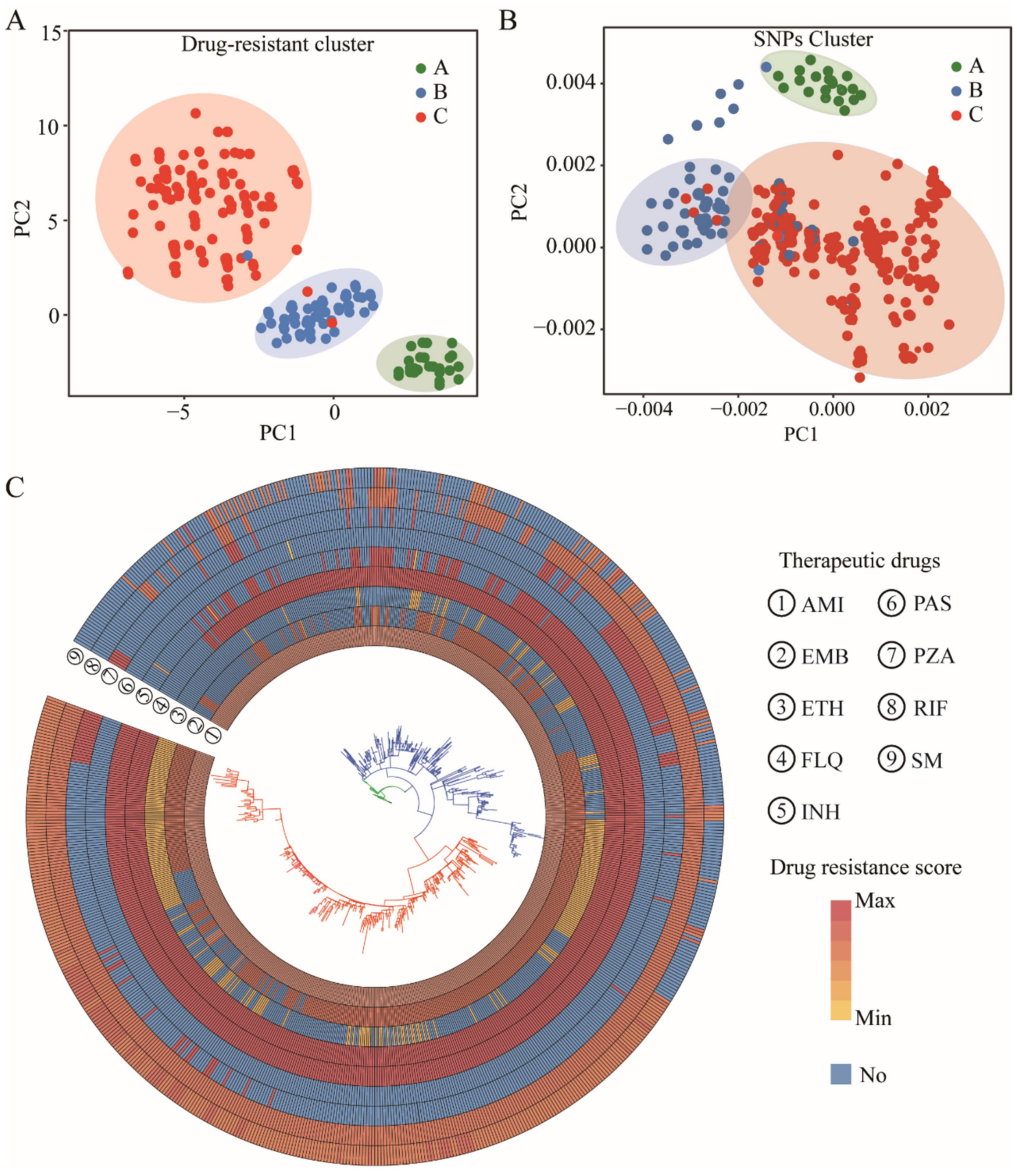
Clustering analysis of *M. tuberculosis* strains. PCA clustering of drug resistance score **(A)** and SNPs **(B)**. The cluster evolutionary tree, the outer circle is a resistance distribution map of nine drugs, and the inner layer is the evolutionary tree itself, with colors showing different cluster groups **(C)**.

**Figure 6:**
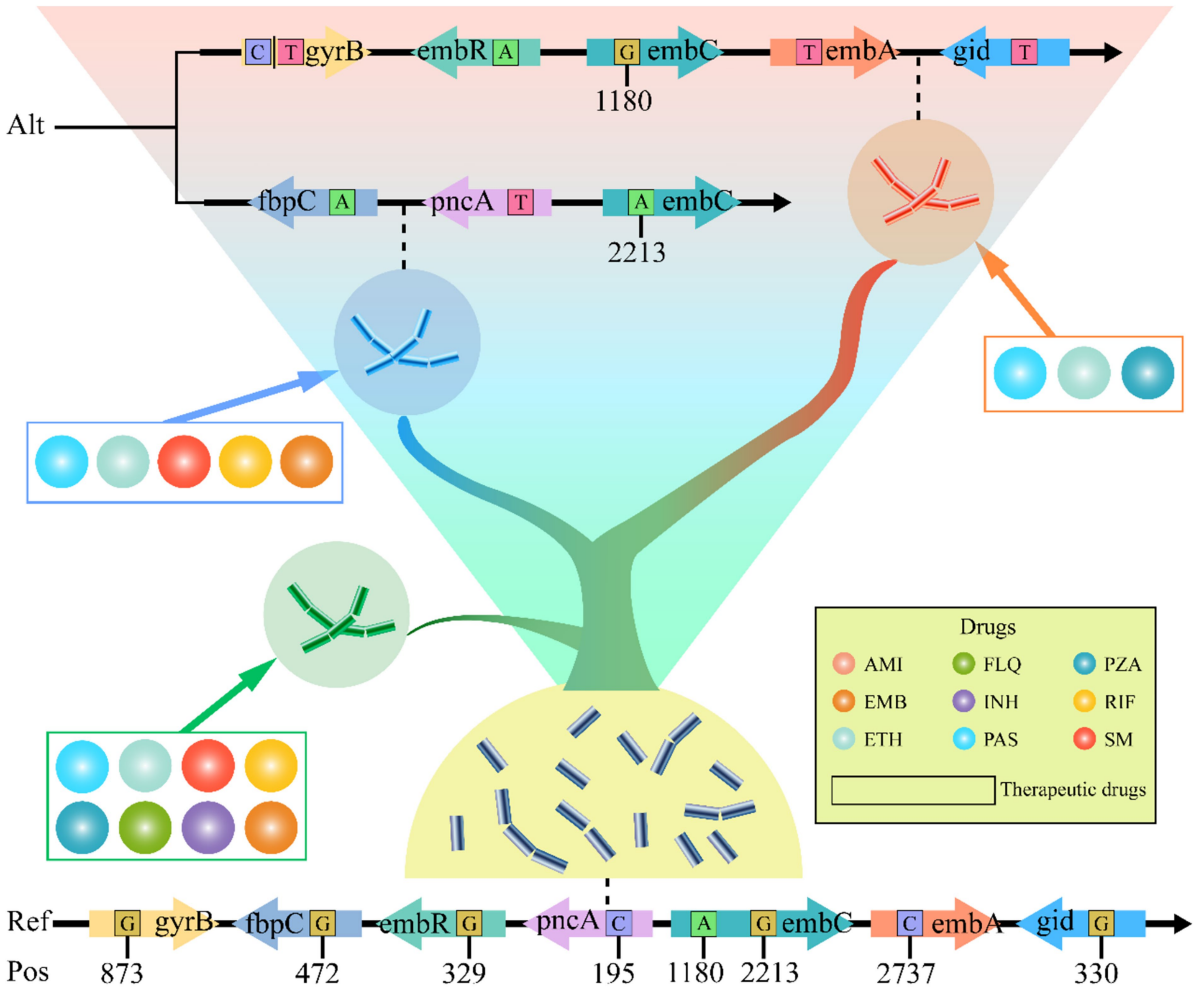
Diagram of *M. tuberculosis* DRMP, with evolutionary levels increasing from bottom to top. Each small circle in the diagram represents an anti-tuberculosis drug, while the drugs enclosed in boxes denote the corresponding therapeutic levels that can be applied. The variant sites in the genes shown are the DRMP. The bases include the alternate allele (Alt) and the reference allele (Ref). The numbers indicate the base positions (Pos) on the gene.

To elucidate the relationship between genetic mutations and drug responses, we conducted an extensive cluster analysis involving 1,140 strains. Utilizing tolerance scores against nine antimicrobial drugs, we discerned three prominent clustering groups through a rigorous examination of internal consistency and clustering effects. This classification was further validated by PCA, which distinctly separated the strains into three coherent groups on the PCA plot (Figure 5A). Intriguingly, when we applied PCA to investigate the association between these drugs and SNPs, a similar pattern emerged. The SNPs were broadly clustered into three subgroups on the PCA plot, suggesting a potential correlation between genetic variations and phenotypic drug responses (Figure 5B). Phylogenetic reconstruction based on multiple genes recapitulated the population structure observed in principal component analysis (PCA) and further demonstrated that allele-specific drug effects were closely aligned with SNP-based clustering patterns (Figure 5C). We presented the drug resistance characteristics of each sample through a heatmap of drug resistance. From early evolutionary stages lacking drug resistance, through intermediate stages where diverse resistance mechanisms emerged, to late stages where resistance stabilized, significant differences in drug resistance existed across clusters. By leveraging these genetic constraints, we established drug-resistance mutation profiles (DRMPs) through the analysis of SNPs within each cluster and identification of those unique to specific clusters. Critically, these DRMPs serve as precise molecular signatures that enable the selection of optimal, cluster-specific drug regimens. This approach facilitates targeted therapy, whether using single agents or tailored drug combinations, thereby maximizing treatment efficacy for distinct *M. tuberculosis* populations (Figures 5C, 6). These analyses underscore the intricate interplay between genetic diversity and drug response, highlighting the potential of customized treatment approaches based on the molecular fingerprints of bacterial strains.

## 4 Discussion

The genetic diversity exhibited by *M. tuberculosis* is a key driver of the emergence of clinical multidrug resistance (Jia et al., 2017; Napier et al., 2020; Shaku and Bishai, 2022), a problem that has long confounded anti-tuberculosis treatment (Farhat et al., 2024; Shu and Liu, 2024). In this study, we employed pan-genomic analysis methods to comprehensively explore the relationship between the evolutionary characteristics of *M. tuberculosis* and its drug resistance, thereby elucidating specific patterns of drug-resistant mutations. These findings provide clearer guidance for the future development of antimicrobial drugs and clinical treatment.

We analyzed over 1,000 *M. tuberculosis* strains from various sources with diverse resistance profiles collected over the past 15 years, examining the diversity in genetic evolution and its correlation with drug-resistant gene mutations. We identified 31 main drug-resistant genes, 94% of which are attributed to the core genes (Figure 3). Further analysis revealed a preference for base mutations closely associated with nonsynonymous mutations at resistance sites, reflecting the adaptive changes in bacteria under drug pressure over the years. These results not only offer new perspectives on the drug-resistant mechanisms of *M. tuberculosis* but also provide a crucial molecular foundation for addressing drug-resistant tuberculosis.

The study shows that starting from drug-sensitive strains, AMI and fluoroquinolones (FLQ) resistance emerged first, followed by cumulative mutations in INH, rifampicin (RIF), and streptomycin (SM) (Figure 5C), indicating more than just simple cross-resistance reported previously. The analysis of evolutionary rates of drug-resistant genes suggests that although most target genes underwent positive selection (Figure 4), such as PAS-targeted thyA; SM-targeted rpsL, gid; and INH-targeted multiple genes, the structural diversity of these target proteins had minimal impact on their function. This provides opportunities for drug-resistant mutations. Interestingly, the tlyA gene under AMI influence underwent passive selection, indicating its conservation and potential lethality of mutations, suggesting that drug target selection should focus on more conserved proteins to minimize resistance. Thus, developing new drugs against resistant strains targeting the tlyA gene remains promising.

Since conventional treatment outcomes are often poor due to variant strains of *M. tuberculosis* (Jang and Chung, 2020; Napier et al., 2020), revising clinical treatment plans and selecting drugs against drug-resistant strains require identification and evaluation of prevalent bacterial strains (Escalante et al., 1998; Lavender et al., 2005; Singh et al., 2020). Previously, this was determined primarily through phenotypic drug susceptibility testing, which involves cumbersome liquid culture screening in microplates and has a long turnaround time. Consequently, the industry has proposed using molecular drug susceptibility to assess and select treatment methods, necessitating a deep understanding of drug-resistant mutation patterns (Domínguez et al., 2023). Although recent studies have used SNP detection methods to assess the drug resistance of *M. tuberculosis*, these mainly focused on single-drug resistance testing (Allix-Béguec et al., 2018; Domínguez et al., 2023). For example, linear probe assays like GenoType MTBDRsl VER 2.0 and cartridge-based methods like Xpert MTB/XDR detect fluoroquinolone resistance (Cao et al., 2021), and Nipro Genoscholar PZA-TB II focuses on the detection of pncA gene mutations related to PZA resistance (Driesen et al., 2018; Willby et al., 2018). However, these methods fall short in comprehensiveness and systematicity. Part of this is due to background noise from random genetic drift, and another part is because drug resistance often results from combined mutations across multiple genes and sites (Ahmad et al., 2016; Chen et al., 2023; Domínguez et al., 2023). Additionally, different drug sensitivity testing (DST) methods may lead to the emergence of discrepant results among isolates (Qadir et al., 2024), which increases the difficulty of fully understanding mutation patterns and evaluating unknown variant strains. Comparative studies on evolutionary patterns under polypharmacy pressure over extended periods can clarify strain characteristics, enabling a more comprehensive drug-resistant assessment of all variant strains (Arnold et al., 2022). Therefore, to provide detailed data support for future molecular drug susceptibility diagnostics, our study reveals the interplay between diversity and drug pressure selection through pan-genome PCA and clustering analysis (Figure 5), and establishes a link between genetic variation and drug-resistant phenotypes based on SNPs differences (Figure 6). This locks in the DRMP, serving as a molecular fingerprint and precise molecular drug susceptibility indicator for resistant strains, aiding in the evaluation of resistant conditions in variant strains (including unknown ones) and determining optimal treatment options, thus facilitating the implementation of precision personalized treatment. Beyond direct diagnosis and treatment guidance, DRMP characterization offers significant clinical and epidemiological value. Clinically, specific mutation patterns may predict resistance-associated fitness costs, influencing *M. tuberculosis* transmissibility and relapse risk. This enables patient stratification for enhanced follow-up or infection control. Epidemiologically, DRMP act as molecular fingerprints for tracking transmission. Clusters sharing rare DRMP signal local outbreaks, while geographically distinct patterns reveal cross-border spread. Pan-genomic DRMP analysis identifies regionally prevalent resistance mechanisms, exposing gaps in local drug regulation or prescribing practices. These insights prioritize targeted surveillance, optimize resource allocation for containment, and inform early-warning systems for emerging threats.

In summary, this study adopts a pan-genomic perspective to comprehensively analyze the correlation between the evolution of *M. tuberculosis* and its drug resistance. The findings suggest that developing new antibiotics targeting certain key and conserved genes can enhance drug sensitivity and decrease the possibility of drug resistance. Moreover, the research reveals a close association between the clustering of SNPs in clinical strains and drug-resistant characteristics, and identifies specific DRMP. This DRMP can serve as precise molecular markers for drug susceptibility, guiding the selection of effective medications and thereby providing personalized treatment options for clinical therapy.

## Data Availability

The original contributions presented in the study are included in the article/Supplementary material, further inquiries can be directed to the corresponding authors.

## Footnotes

^1^ https://www.ncbi.nlm.nih.gov/sra

^2^ https://www.ncbi.nlm.nih.gov/datasets/genome/?taxon=1773

^3^ https://busco-data.ezlab.org/v5/data/lineages/

^4^ https://github.com/dbespiatykh/TB-gen

^5^ https://github.com/Ankitapal1995/TB-AMRpred

^6^ https://github.com/avkitex/tbAnnotator
